# Green chromatographic methods for determination of co-formulated lidocaine hydrochloride and miconazole nitrate along with an endocrine disruptor preservative and potential impurity

**DOI:** 10.1186/s13065-023-01065-3

**Published:** 2023-11-08

**Authors:** Esraa S. Ashour, Maha A. Hegazy, Amal M. Abou Al-Alamein, Ghada M. El-Sayed, Nermine S. Ghoniem

**Affiliations:** Analytical Chemistry Department, Faculty of Pharmacy, Cairo University, Kasr-El-Aini, Cairo, 11562 Egypt

**Keywords:** Chromatographic separation, Lidocaine, Miconazole, Toxic impurity, Endocrine Disruptor, Greenness assessment

## Abstract

Recently, green analytical chemistry (GAC) is a key issue towards the idea of sustainability, the analytical community is focused on developing analytical methods that incorporate green chemistry principles to minimize adverse impacts on the environment and humans. Herein, we present 2 sustainable, selective, and validated chromatographic methods. Initially, lidocaine hydrochloride (LDC) and miconazole nitrate (MIC) with two preservatives; methyl paraben (MTP) and saccharin sodium (SAC) were chromatographed via TLC–densitometric method which employed ethyl acetate: methanol: formic acid (9:1:0.1, by volume) as the mobile phase with UV detection at 220.0 nm, good correlation was obtained in the range of 0.3–3.0 µg/band for MIC and LDC. Following that, RP-HPLC was successfully applied for separating quinary mixture of LDC, MIC, MTP, SAC along with LDC impurity; dimethyl aniline (DMA) using C18 column, and a gradient green mobile phase composed of methanol and phosphate buffer (pH 6.0) in different ratios with a flow rate 1.5 mL/min and UV detection at 210.0 nm, linearity ranges from 1.00 to 100.00 µg/mL for MIC, 2.00–100.00 µg/mL for LDC and 1.00–-20.00 µg/mL for MTP and DMA. No records to date regarding the determination of the two drugs, besides MTP and DMA. The proposed methods were validated according to the ICH guidelines and applied successfully to the analysis of the compounds. The methods' results were statistically compared to those obtained by applying the reported one, indicating no significant difference regarding both accuracy and precision. The methods' greenness profiles have been assessed and compared with those of the reported method using different assessment tools.

**Graphical Abstract:**

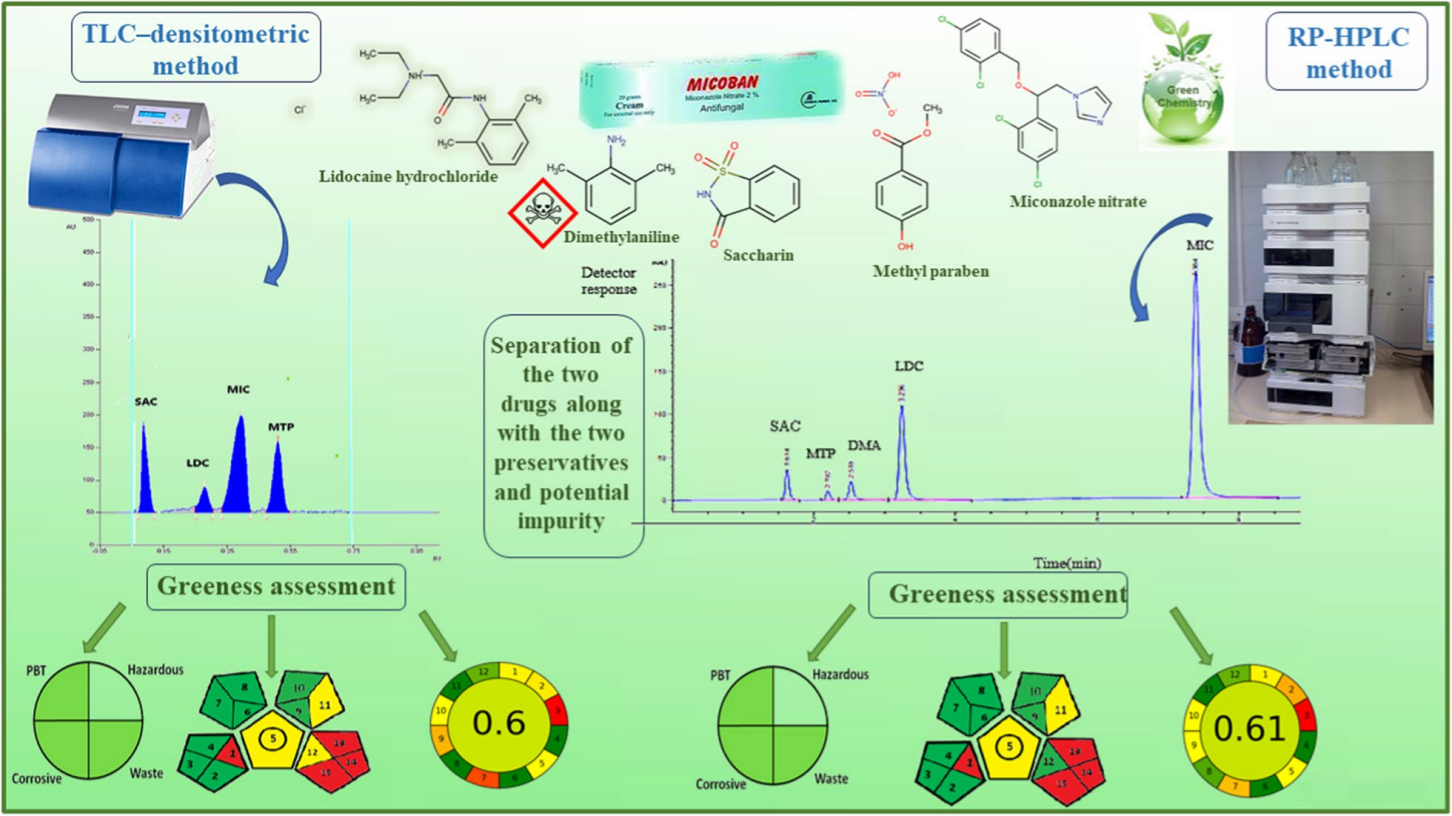

## Introduction

**Fig. 1:**
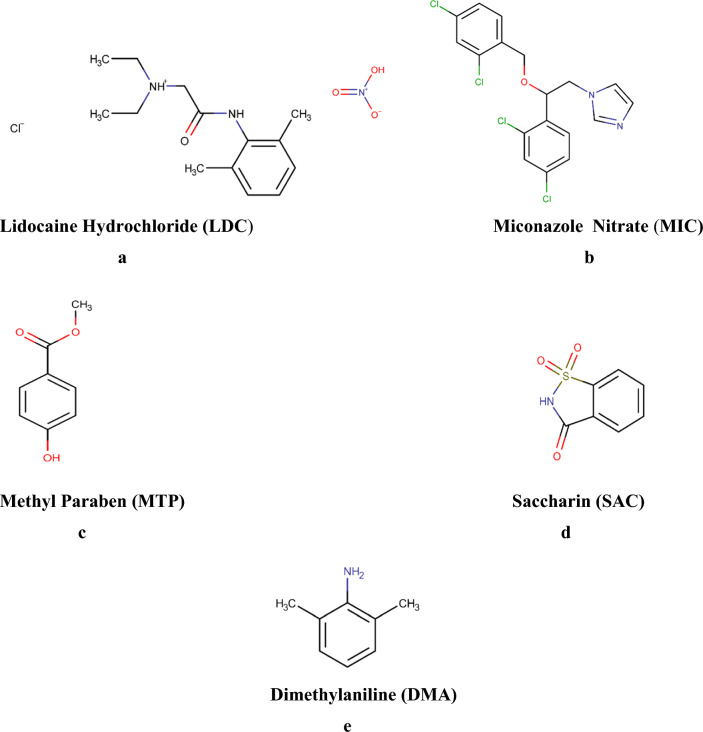
Chemical structures of lidocaine hydrochloride, miconazole nitrate, methyl paraben, saccharin and dimethylaniline. **a** Lidocaine Hydrochloride (LDC). **b** Miconazole Nitrate (MIC). **c** Methyl Paraben (MTP). **d** Saccharin (SAC). **e** Dimethylaniline (DMA)

Lidocaine hydrochloride (LDC) is chemically designed as 2-(diethyl amino)-N-(2,6-dimethylphenyl) acetamide hydrochloride, Fig. 1a, it is a local anesthetic, LDC combination can be directly applied to the skin and mucous membranes to make the area numb via suppressing the inward Na.^+^current upon depolarization at the sodium ionophore, which inhibits the axonal action potential propagation, it usually begins functioning within a few minutes and lasts for 30 min to three hours when used for local anaesthesia or in nerve blocks [1, 2]. It is on the WHO list of essential medicines [3] Miconazole nitrate (MIC) is 1-[(2RS)-2-[(2,4-dichlorobenzyl) oxy]-2-(2,4-dichlorophenyl) ethyl)]-1H-imidazole nitrate, Fig. 1b, MIC is an imidazole antifungal, it’s one of the most extensively utilized azoles on the market [1]. MIC has dual mechanisms of actions, the first mechanism involves the suppression of ergosterol production and the other one includes the inhibition of peroxidases, which results in a buildup of peroxide inside the cell and eventually causes cell death [4]. Its combination with LDC, is marketed as antifungal oral gel used for treatment of candidal infection of the gastrointestinal tract and the oropharyngeal cavity [5]

Methyl paraben (MTP) is methyl 4-hydroxybenzoate, Fig. 1c. Parabens are utilized extensively in a variety of industries, as preservatives and antimicrobial agents [6, 7]. Parabens are effective substances, but their widespread use is under debate among a number of organizations and the scientific community due to their reputation of being harmful to several aquatic creatures, in addition, these substances have a number of health concerns, because of the endocrine disrupting impact, including breast cancer and reproductive system issues, added to some publications claim that they are carcinogenic substances as they can exhibit estrogenic and antiandrogenic activity, these molecules have been linked to cancers, specifically breast tumors and male infertility [8]. Parabens also showed the ability to be passed from mother to child via trans placental transmission, the impact of prenatal paraben exposure on thyroid function in newborns and birth weight has been studied and also, several research studies have suggested that parabens are to blame for persistent urticaria or angioedema, as a result, parabens are now being considered a possible health danger [9]. This type of emergent chemical is becoming more prevalent in ecosystems causing harm to the environment, wildlife, and even people. The presence of this sort of chemical in ecosystems is increasing the environment, animals, and even humans [10]. Thus, antimicrobial preservatives as parabens in medical products need to be justified and they must be controlled during the analysis and batch release and there are acceptance criteria that have to be fulfilled. MTP is utilized in oral preparations at concentrations ranging from 0.015 to 0.2% [9].

Saccharin sodium (SAC) chemically named as 2H-1λ6,2-benzothiazol-1,1,3-trione (SAC), Fig. 1d, SAC is a pharmacologically inactive substance, it is used as a non-nutritive sweetener in oral pharmaceutical formulations, separation of SAC was crucial, as it’s extracted with the active ingredients in the oral gel. Dimethylaniline (DMA) the major impurity of LDC chemically named as N, N-Dimethylaniline, Fig. 1e, it is a pharmacologically inactive metabolite, regarded as a major degradation product of LDC [1], Hence, it is crucial to be identified in pharmaceutical formulations, as it is absorbed through the mucosa of the mouth and the skin affecting organs such as the kidneys, liver, and heart [11]. The BP claimed that the impurity limit was 0.01 percent [1]

A review of the literature in hand revealed a number of approaches which were applied to determine the studied drugs separately or combined with other compounds using various analytical techniques. For LDC determination either alone or in combination with other drugs chromatographic [12, 13] capillary electrophoretic [14] and spectrophotometric [15, 16] have been reported, in addition, for the estimation of LDC with DMA, spectrophotometric [17] voltammetric, [18] and electrochemical approaches [19] approaches have been published. MIC was determined individually or combined with other compounds by stability indicating High Performance Thin Layer Chromatography (HPTLC) [20, 21], High Performance Liquid Chromatography (HPLC), Chemometrics-assisted UV spectrophotometry [22, 23] and voltammetry [24] On the other hand, for the determination of MTP, stability indicating- HPTLC [25], electrochemical [26] and spectrophotometric [27] methods have been published, besides for SAC determination HPLC [28, 29] methods have been reported. According to what we know, there was only one reported method, which is HPLC–DAD for the determination of LDC and MIC in their co-formulated gel dosage form [30].

After reviewing the literature, no records so far for the separation and the determination of the binary mixture of LDC and MIC along with MTP and DMA in presence of SAC.

Green analytical chemistry (GAC) is a novel approach that emerged in 2000s [31]. This emerging discipline is concerned with establishing analytical processes that limit the use of harsh chemicals or reagents while also increasing analyst and environmental safety [32–34]. Significant advancements in methodological tools have been developed in latest years to minimize the negative impacts of analytical methods [35, 36]. Following GAC's principles and recommendations is essential to achieving balance between attaining maximum results and minimizing the environmental issues related with analytical methodologies [37]. The word SIGNIFICANCE represents the GAC principles [31]:

The approaches for evaluating an analytical method's greenness should be dependable assessment tool. A tool like this should be evaluated, assessed, and used as the primary parameter for building a green analytical approach [37]. These include, for example, National Environmental Methods Index (NEMI), this is regarded as the most traditional form of evaluation [38], Green Analytical Procedure Index (GAPI) [39] and the recently established “Analytical Greenness metric” (AGREE)[40–42].

Hence, In the current work, authors target to develop green, simple, and rapid TLC method for determination of LDC and MIC, which has the advantages of being accurate, selective, and quick for routine quantitative analysis, also it minimizes sample preparations, laboratories consumption and cost materials [43, 44], it is commonly utilized in the pharmaceutical industry's research and quality-control labs [45]. Considering that it was necessary to identify and quantify the hazardous impurity related to LDC that might be present in the stated combination and also the endocrine disruptor MTP, RP-HPLC method was a step forward as it managed to separate and determine LDC, MIC, and MTP in their gel formulation along with DMA in presence of SAC with greater precision, accuracy, and sensitivity.

## Experimental

### Instrumentation

#### TLC method

CAMAG TLC Scanner model 3S/N 1302139 operated with winCATS software (CAMAG, Switzerland) was used for scanning, CAMAG TLC autosampler Linomat (CAMAG, Muttenz, Switzerland) with a 100.0 µL microsyringe. Aluminum TLC plates precoated with 0.25 mm silica gel with florescent indicator F_254_ size 20 × 20 cm (Merck, Germany),

#### RP-HPLC method

An HPLC system (model 1260 infinity series; Agilent, Germany) consists of an Agilent quaternary pump (model G1311C, serial No. DEAB816766) with different flow rates, equipped with a photodiode array detector (model G1315D, Agilent, Germany, serial No. DEAAX06967) and a manual injector (model G1328C, serial No. DEABG03628) with 20-µL injection loop, and the system is operated by Agilent ChemStation software. Waters X Select® CSH^TM^C18 column (250 mm × 4.6 mm I.D, particle size 5 µm) was used as stationary phase. A Soniclean 160 T sonicator (Soniclean, Thebarton, Australia) was used for extraction of drugs from pharmaceutical dosage form. pH-meter (Jenway model 3505, UK) was also used.

### Materials and reagents

#### Pure standards

Miconazole, methyl paraben, lidocaine, and saccharin their purities were certified to be 99.5%, 98%, 99.9%, and 98% respectively, were kindly provided by Amriya Pharmaceutical Industries (Alexandria, Egypt). 2, 6-Dimethylaniline was supplied by Sigma-Aldrich (Egypt), with a purity of 99.9%.

#### Pharmaceutical formulation

Micoban® oral gel (25 mg/6.6 mg), batch no 5875003, labelled to contain 2.5% (w/w) MIC and 0.66% (w/w) LDC per gram, as well as the inactive ingredients: MTP and SAC, manufactured by Amriya Pharmaceutical Industries, Alexandria, Egypt and obtained from the local Egyptian market.

#### Chemicals and reagents

The chemicals utilized were of analytical reagent grade, and the solvents used were of an HPLC grade; Methanol, sodium hydroxide (Sigma Aldrich, Darmstadt, Germany), potassium dihydrogen phosphate (E. Merck, Darmstadt, Germany), ethyl acetate, formic acid (El-Nasr Pharmaceutical Chemicals Co., Cairo, Egypt) and double distilled deionized water (Otsuka, Cairo, Egypt). Phosphate buffer solution pH 6.0 (made by dissolving 2.72 g of potassium dihydrogen phosphate in 1 L of double distilled deionized water, its pH was adjusted with 10% sodium hydroxide to pH 6.0) [1].

#### Stock standard solutions

##### TLC method

Stock standard solutions for LDC, MIC, MTP and SAC were prepared in methanol, by accurately weighing and transferring 10.0 mg of each standard into a 10-mL volumetric flask to attain 1.0 mg/mL final concentrations for each drug individually.

##### RP-HPLC method

Stock standard solutions for LDC, MIC, MTP, DMA and SAC were prepared, each at a concentration of 1.0 mg/mL in methanol, by accurately weighing and transferring 25.0 mg of each of the pure standards to 25-mL volumetric flask. Working standard solutions (100.00 µg/mL) were prepared by transferring 10.0 mL of stock standard solution separately into 100- mL volumetric flasks and completing to the mark with methanol.

### Procedures

#### Chromatographic conditions

##### TLC method

Samples were individually applied in triplicates as bands onto TLC plates, the band length was 3.0 mm. The bands were placed 15 mm from the plate's bottom edge and at intervals of 10 mm. The chromatographic chamber was previously saturated with the developing system [ethyl acetate: methanol: 0.1% formic acid (9:1:0.1, by volume] for about 30 min at room temperature. After then, the plates were developed by ascending chromatography (for 7.5 cm). The plates were air dried after development, then they were scanned at 220.0 nm.

##### RP-HPLC method

Using a gradient mobile phase of methanol (A) and phosphate buffer pH 6.0 (B), which was ultrasonically degassed before injection, the separation was accomplished in the following manner: starting at (80:20, v/v) for 2 min, ramping up to (90:10, v/v) for the following 2 min, this ratio was kept till the end of the run and 3 min of reconditioning was applied in between runs. We performed the chromatographic separation on Waters X Select® CSH^TM^C18 column (250 mm × 4.6 mm I.D, particle size 5 µm), the flow rate was 1.5 mL/min, and detection wavelength was 210.0 nm, both mobile phase and samples were filtered through 0.24 m filters. The injection volume was 20.0μL, all measurements were conducted at ambient temperature.

##### Construction of calibration curve

###### For TLC

Accurately measured aliquots of LDC, and MIC equivalent to (0.3–3.0 µg/band) for both drugs were transferred from their stock standard solutions (1.00 mg/mL) and applied onto three different TLC plates in the form of bands using with a 100.0 µL microsyringe. After the plates were developed using the previously mentioned optimized conditions, they were air dried, bands were visible at 254 nm under a UV lamp, and the chromatogram was scanned at 220.0 nm. Calibration curves illustrating the relation between the mean integrated peak area and the corresponding concentration of each of LDC, and MIC (0.3–3.0 µg/band), were plotted.

###### For HPLC

Different volumes equivalent to 1.00 –100.00 µg /mL for MIC, 2.00 –100.00 µg /mL for LDC and 1.00 -20.00 µg /mL for MTP and DMA, separately, were accurately transferred from their stock solutions into 10-mL measuring flasks, diluted to the mark with a solution of methanol–phosphate buffer pH 6.0 (50:50, v/v). In triplicate, a 20.0 µL of these solutions were injected into the column and chromatographed using the optimized conditions described earlier. The calibration curves were created by plotting the average area under the peak against the relevant concentration and then computing the regression equations.

### Application to pharmaceutical formulation

Into a 50-mL beaker, a 1.0 g of the oral gel preparation was accurately weighed and sonicated for 15 min in methanol, filtered into 100-mL volumetric flask, and the volume is completed with methanol. For TLC method 10.0 μL was applied onto TLC plates. For RP-HPLC method 2.0 mL aliquots claimed to contain (50.00 µg/mL MIC and 13.20 µg/mL LDC) were transferred to 10-mL measuring flasks and completed with a solution of methanol–phosphate buffer pH 6.0 (50:50, v/v). The prepared solutions were chromatographed under the chromatographic conditions described above and the concentrations were calculated from the corresponding regression equations.

## Results and discussion

A survey of the literature revealed that, the only reported method for determination of LDC and MIC in their gel dosage form is HPLC–DAD and there was no reported technique available for simultaneous MTP and DMA determination besides the two active ingredients LDC and MIC in laboratory mixture and dosage form. Initially, the development of the proposed TLC method is used for quantification the two drugs in presence of the two inactive ingredients: MTP, and SAC.

Given that the effect of parabens on people, animals, and ecosystems is a debatable issue and that DMA has negative health effects, there is an increasing need for DMA and MTP quantification. As a result, we present an eco-friendly, precise, sensitive, and gradient RP-HPLC technique to assess the opportunities presented by this separation technique for determination the toxic MTP and DMA besides LDC and MIC.

### Methods Development and optimization

#### Optimization of the TLC method

##### Developing system

There have been attempts at various development systems with various ratios for the maximum separation of LDC, MIC, MTP and SAC. Initially, butanol: water: acetic acid (6:2:2, by volume) system was tried but analysis time was too long. Second, a good separation was accomplished using chloroform, ethyl acetate, and toluene (5:4:3, by volume), but still we search for a green system, hence another system composed of acetone: ethyl acetate (3:7, v/v) was tried and also, a satisfactory separation was obtained. Eventually in order to extend our search for terms of greenness, different ratios of methanol: ethyl acetate were attempted, and we concluded that ethyl acetate: methanol (9:1, v/v) was the best ratio, also we found that the pH of the system affects the resolution of the two bands of LDC and MIC, so formic acid is added. In conclusion [ethyl acetate: methanol: 0.1% formic acid (9:1:0.1, by volume] was the ideal developing system for the optimum separation with the maximum greenness.

##### Scanning wavelength

**Fig. 2:**
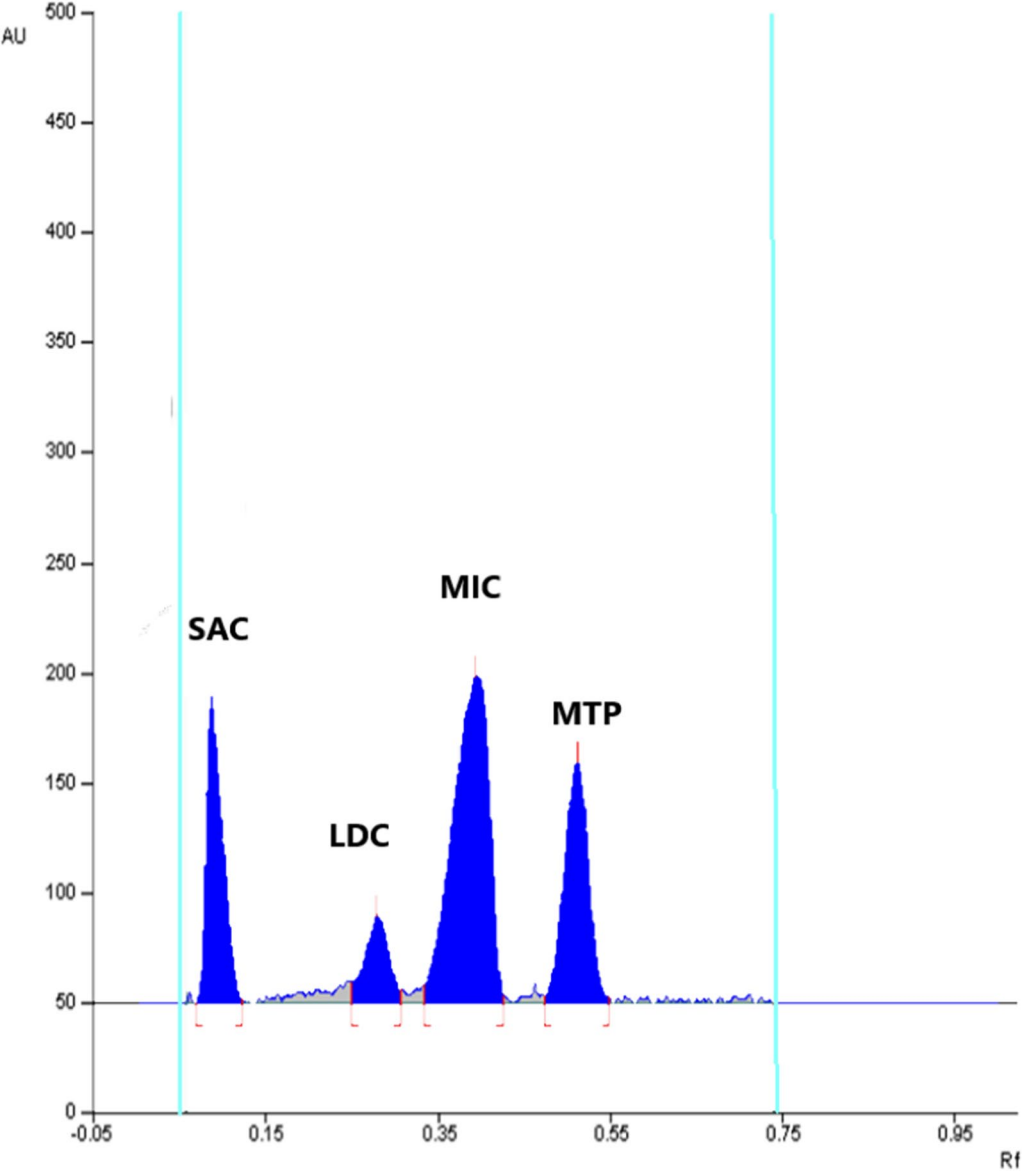
TLC Chromatogram of laboratory prepared mixture containing 0.50 µg/band SAC (Rf = 0.12 ± 0.01), 1.20 µg/band LDC (Rf = 0.30 ± 0.01), 1.00 µg/band MIC (Rf = 0.42 ± 0.01) and 0.50 µg/band MTP(Rf = 0.54 ± 0.01) using a mobile phase consisting of ethyl acetate: methanol: formic acid (9:1:0.1, v/v/v) and detection was performed at 220.0 nm

For attaining good sensitivity of LDC, MIC, MTP and SAC with minimal noise, three different wavelengths were tried (210.0, 220.0, and 230.0 nm). Satisfactory results were obtained by using the wavelength 220.0 nm which gave sharp and symmetrical peaks for the four drugs with high sensitivity and minimum noise as shown in, Fig. 2.

##### Optimization of the RP-HPLC method

**Fig. 3:**
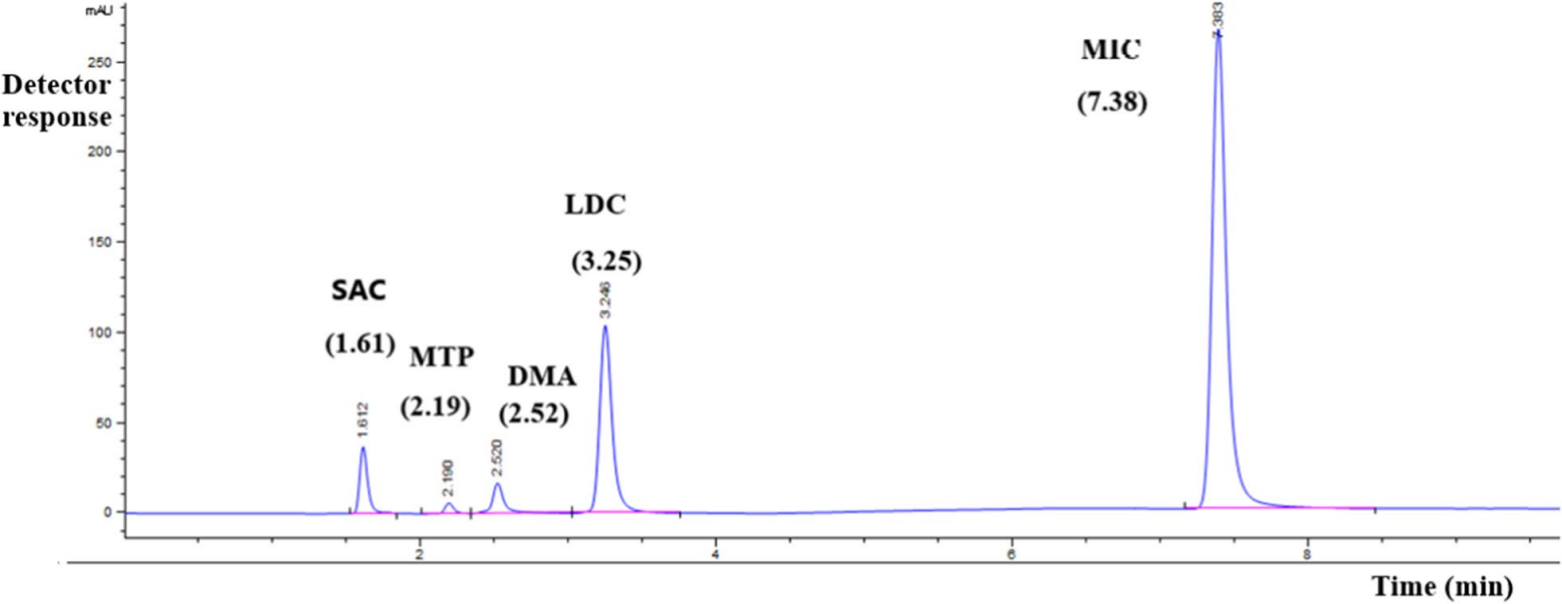
HPLC chromatogram of a resolved mixture of 0.50 μg/mL SAC, 1.00 μg/mL MTP, 1.00 μg/mL DMA, 50.00 μg/mL LDC and 50.00 μg/mL MIC, on a 25.0 cm X-select column, using gradient mobile phase composed of methanol and phosphate buffer (pH 6.0) in different ratios

There were several attempts for the simultaneous separation of the five components. By trying methanol and phosphate buffer with isocratic elution in different ratios 90: 10, v/v and 85: 15, v/v, resolution was less than the accepted limit. Also, in case of isocratic elution, by trying 80: 20 methanol and phosphate buffer ratio, longer time of analysis was obtained, so gradient elution was employed. Different flow rates were tried, 1.0 mL/min and 1.2 mL/min, longer run time was obtained. The optimal flow rate was 1.5 mL/min, which yielded lower retention time for all analytes while maintaining acceptable peak resolution. Finally, a good separation was accomplished by the above-mentioned optimum conditions. The suggested system allows good baseline separation with optimal resolution. A chromatogram of SAC, MTP, DMA, LDC and MIC is shown in Fig. 3, retention times were 1.61 min, 2.19 min, 2.52 min, 3.25 min and 7.38 min, respectively.

#### Composition of the mobile phase and elution mode

One of the goals of this work was to develop an approach that was environmentally beneficial. As a result, we consider two factors: solvent safety and waste minimization. If a solvent-free method is not viable, the green option is solvent reduction or the replacement of organic solvents with ‘‘green solvents’’ [46]. Environment-friendly solvents include methanol, ethanol, ethyl acetate, heptane, and hexane. Acetonitrile, on the other hand, is not environmentally friendly [47]. The findings also revealed that ethanol/water and methanol/water mixes are less harmful to the environment than pure alcohol. Solvents are ranked in descending order of greenness depending on a variety of factors. Water, for example, ranks first, followed by ethanol and acetone, whereas carbon tetrachloride and benzene rank last [48]. Because ethanol/water mixtures have a higher viscosity than methanol/water mixtures, they are more viscous at ambient temperature and high backpressures result from such high viscosity, methanol is the substance of choice in RP-HPLC and TLC methods, since the prior published LC technique [30] employed acetonitrile as the mobile phase, also buffer was used instead of water to attain shorter analysis time without sacrificing the greenness of the mobile phase. Additionally, in TLC method ethyl acetate is chosen instead of chloroform and toluene which are not environmentally friendly.

#### Method validation

The method's system suitability parameters were determined [49] showing acceptable resolution, peak symmetry, and selectivity (Table 1). The suggested methods were validated in compliance with ICH Q2 (R1) guideline [50].

**Table 1:** System suitability parameters of the proposed RP-HPLC and TLC methods

Parameter	RP-HPLC						TLC					Reference values	
	SAC	MTP	DMA		LDC	MIC	SAC	LDC	MIC		MTP	HPLC	TLC
Retention time (t_R_) / Retardation factor	1.60	2.19	2.52		3.25	7.38	0.12	0.30	0.42		0.54	–	
Tailing factor (T)	1.05	1.00	0.88		0 .95	1.09	1.18	1.00	0.79		1.00	T = 1 for a symmetric peak	T = 1 for a symmetric peak
Capacity factor (K`)	1.30	2.12	2.60		3.63	9.55	7.33	2.33	1.38		0.85	1–10	The higher the capacity factor, the smaller the Rf
Resolution factor (R_s_)	10.37	3.85	2.21		3.20		1.23	1.00	1.02			R_s_ > 2	Rs > 1
Selectivity factor (α)	2.63	1.63	1.23		1.40		3.15	1.69	1.62			α > 1	α > 1
Number of theoretical plates (N)	1840	3401	4508		1863	3492						Increase with the efficiency of the separation	
HETP	0.136	0.074	0.055		0.134	0.072						The smaller the value, the higher the column efficiency	

#### Range and linearity

Under optimal conditions, five concentrations in the range of (0.3–3.0 µg/band) for LDC, and MIC were analyzed in triplicate for TLC method. Seven concentrations were determined in triplicates in the range of 1.00–100.00 µg/mL for MIC and 2.00–100.00 µg /mL for LDC, five and six concentrations (1.00–20.00 µg/mL) for MTP and DMA respectively for RP-HPLC method were analyzed under the specified chromatographic conditions for each method. Linear correlations were observed between the average peak areas and corresponding concentrations (Table 2).

**Table 2:** Assay validation parameters of the proposed RP-HPLC and TLC methods

Parameter	RP-HPLC				TLC	
	LDC	MIC	MTP	DMA	LDC	MIC
Linearity						
Range	2.00–100.00 µg/mL	1.00–100.00 µg/mL	1.00–20.00 µg/mL	1.00–20.00 µg/mL	0.30–3.00 µg/band	0.30–3.00 µg/band
Intercept	46.441	4.4143	−1.4165	65.621	349.67	1217.5
Slope	64.967	66.568	89	125.33	1120.5	4718
Correlation coefficient (r)	0.9999	0.9999	0.9999	1	1	1
Precision(± %RSD)						
Repeatabiliy^a^	0.881	0.869	0.50	1.133	0.862	1.228
Intermediate precision^b^	1.193	1.067	0.531	1.23	1.866	1.72
Accuracy^c^ Mean % RSD	101.04 0.782	101.31 0.936	99.62 1.371	99.91 1.302	101.1 1.658	100.72 0.667
Specificity^d^ Mean % RSD	100.16 1.078	100.37 1.71	99.91 0.856	99.73 0.84	99.63 0.964	100.1 0.547
LOD^e^	–	–	0.231	0.084	–	–
LOQ^e^	–	–	0.70	0.255	–	–
Robustness parameter^f^ (%RSD)	1.054	1.247	1.28	1.423	0.765	1.427

^a^Repeatability was calculated as %RSD of three different concentrations of LDC and MIC within the same day for TLC method and three different concentrations of MIC, LDC, MTP and DMA for RP-HPLC method

^b^ Intermediate precision replication of the same concentrations of the examined drugs was carried out on 3 successive days and %RSD values were calculated

^c^ Accuracy was tested by calculating the average recovery of Three pure samples for LDC and MIC for TLC and Five pure samples for MIC, LDC, MTP and DMA for RP-HPLC method in triplicates

^d^ Specificity was tested by calculating average determinations of mixtures containing LDC and MIC for TLC method and the two drugs, MTP and DMA for RP-HPLC method in various ratios

^e^ LOD and LOQ were calculated from the standard deviation (s) of the regression residuals and the slope of the calibration curve (S) according to the following equations: LOD = 3.3(s/S) and LOQ = 10(s/S)

^f^ The Robustness for TLC method, it is assessed by applying small changes in the wavelength by 1 nm and saturation time ± 5 min. While for HPLC method, small changes in the ratio of pH by 0.05, flow rate ± 0.05 mL /min and the change in wavelength (210 ± 1 nm) are applied and %RSD values were calculated

#### Accuracy

Three pure samples (1.20, 1.40 and 1.50 µg/band) for LDC, and (0.90, 1.20 and 1.50 µg/band) for MIC were determined in triplicates for TLC method and five pure samples (7.00, 20.00, 40.00, 60.00 and 80.00 µg/mL) for MIC and LDC, and (3.00, 6.00, 12.00, 14.00 and 17.00 µg/mL) for MTP and DMA were analyzed in triplicates for RP-HPLC method to ensure the accuracy of the suggested techniques. The % recoveries were calculated using the concentrations obtained from the relevant regression equations. The percentage recoveries obtained indicated that the suggested methods were accurate (Table 2).

#### Precision

Repeatability (intra-day precision), three different concentrations of LDC (0.50, 0.70 and 2.00 µg/band), and (0.70, 1.00 and 3.00 µg/band) for MIC were determined in triplicates on three successive times within the same day for TLC method and three different concentrations of MIC and LDC (30.00, 50.00 and 70.00 µg/mL) and (5.00, 10.00, 15.00 µg/mL) for MTP and DMA for RP-HPLC method and the results are displayed in (Table 2) indicating good precision with small values of percentage relative standard deviation (% RSD).

Intermediate precision (inter-day precision), on three consecutive days, replication of the same three concentrations of the examined drugs was carried out. The % RSD values were shown to be minimal, indicating that the data was reasonably repeatable (Table 2).

#### Specificity

Mixtures containing LDC, and MIC for TLC method, in addition, the two drugs, MTP and DMA for RP-HPLC method in various ratios were used to determine specificity under the previous conditions mentioned. Specificity was monitored by the changes of peak areas and then calculating the %RSD. Table 2 shows that the proposed methods have satisfactory results.

#### Robustness

Robustness refers to a system's ability to remain unaffected by slight changes in method parameters. For TLC method, changing the wavelength by 1 nm and saturation time ± 5 min (Table 2). For RP-HPLC method, changing in the ratio of pH by 0.05, flow rate ± 0.05 mL/min and the change in wavelength (210.0 ± 1 nm). Response was monitored by the changes of peak areas and then calculating the %RSD which showed that small deliberate changes in the tested parameters had no effect on the methods' stability Table 2.

#### Application to pharmaceutical formulation

The suggested methods were used to determine MIC and LDC in their gel dosage form (Micoban® oral gel). Applicability of the proposed procedures for determining these chemicals in their formulations on a regular basis is confirmed by the percentage of recoveries being within the specified range with the application of the standard addition technique for the RP-HPLC method, the accuracy of the proposed techniques is further evaluated (Table 3).

**Table 3:** Analysis of LDC and MIC in their dosage form by the proposed TLC and RP-HPLC methods with the application of standard addition technique for RP-HPLC method

Drug	TLC-Densitometric		RP-HPLC			
	Claimed Concentration µg/ band	Recovery %^a^	Claimed concentration (µg mL^−1^)	Recovery%^b^	Added concentration (µg mL^−1^)	Recovery%
MIC	2.50	98.80 ± 0.4	50.00	100.57 ± 1.301	15.00	98.20
					30.00	100.13
	Mean ± SD				45.00	101.84
			Mean ± SD			100.06 ± 1.821
LDC	Claimed Concentration µg/ band	Recovery %^a^	Claimed concentration (µg mL^−1^)	Recovery%^b^	Added concentration (µg mL^−1^)	Recovery%
	0.66	99.49 ± 0.878	13.20	99.91 ± 1.334	8.00	99.50
					16.00	101.75
	Mean ± SD				24.00	99.25
			Mean ± SD			100.17 ± 1.349

^a^average of three determinations

^b^average of six determinations

#### Statistical analysis

The results of the suggested RP-HPLC method for the analysis of pure samples of MIC and LDC and those obtained by the analysis of MIC and LDC in their dosage form by the TLC method were compared statistically to the results obtained by the reported HPLC–DAD method [30]. The calculated t and F values were lower than the tabulated ones indicating that there is no significant difference between the suggested methods and the reported one (Table 4).

**Table 4:** Statistical comparison between the proposed methods and a reported HPLC method for the determination of LDC and MIC

Value	Proposed TLC method		Reported method ^a^ (Dosage form)		Proposed RP-HPLC method		Reported method (Pure form)	
	LDC	MIC	LDC	MIC	LDC	MIC	LDC	MIC
Mean	99.49	98.8	98.89	99.24	101.04	100.94	100.53	98.9
SD	0.878	0.4	1.438	0.86	0.79	1.741	1.66	1.048
N	3	3	5	5	5	5	5	5
Variance	0.877	0.4	2.0674	0.7397	0.624	3.031	2.756	1.098
Student’s t-test ^b^ (2.447)	0.636	0.761			0.620	2.245		
F value^b^ (19.25)	2.36	1.85			4.42	2.76		

^a^Gradient HPLC–DAD stability indicating determination of miconazole nitrate and lidocaine hydrochloride in their combined oral gel dosage form

^b^Figures in parentheses are the corresponding tabulated values at p = 0.05

#### Assessment of the proposed method’s greenness

The suggested methods' greenness profile was assessed and graded in contrast to the published technique using the following three assessment tools:

#### National environmental method index (NEMI)

NEMI is a tool used to assess the analytical procedures' environmental sustainability. Using a symbol divided into four quadrants, despite the fact that it is the least accurate tool for assessing the method's greenness [38]. The profile requirements are described by four keywords: PBT, hazardous, corrosive, and waste are all represented by the four quadrants, 1-PBT is persistent, bio-accumulative, and toxic. If the Environmental Protection Agency's Toxic Release Inventory (EPA-TRI) does not classify the chemicals as PBT, the appropriate quadrant is colored green [51]. 2-The chemicals utilized aren't dangerous, thus they're not included on the TRI list [52]. 3-the medium's pH ranges from 2 to 12; 4- the waste produced is less than 50 g. For the suggested methods and the published one, we designed the NEMI pictograms (Table 5). The reagents and solvents utilized aren't PBT. The methanol used is classified as hazardous by the TRI list. Because the pH is 6.0, the method is regarded as non-corrosive. The amount of waste produced is less than 50 g for RP-HPLC and TLC methods. The published methodology also had one non-shaded quadrant that related to hazardous quadrant.

**Table 5:** Comparison of the greenness profiles of proposed methods and the reported one using NEMI, GAPI, and AGREE tools

Chromatographic condition	NEMI tool	GAPI tool	AGREE tool
Proposed RP-HPLC method The method was developed by using Waters X Select® CSHTMC18 column and a gradient mobile phase composed of methanol and phosphate buffer (pH 6) in different ratios. The flow rate was 1.5 mL/min and detection limit was 210 nm			
Proposed TLC method The method was developed by using aluminum TLC plates precoated with silica gel 60F254 as the stationary phase, mobile phase was consisting of ethyl acetate: methanol: formic acid (9:1:0.1, v/v/v) and detection was performed at 220.0 nm			
Reported metho The method was developed using C8 column with gradient elution of the mobile phase composed of 0.05 M phosphoric acid and acetonitrile, at a flow rate of 1 mL/min and the multiple wavelength detector was set at 215 nm			

#### Green analytical procedure index (GAPI)

The GAPI assessment that has been developed could be a useful semi quantitative tool for lab research and education [39]. It is used to evaluate the overall greenness of an analytical procedure [53]. It covers 15 different aspects of sample preparation and collection, as well as the safety and health implications of the chemicals and substances employed, waste management, and equipment [54]. For each step, GAPI utilizes a three-color scale: ranging from green to yellow to red just like traffic lights, where green denotes a safe technique and red denotes operations that are not environmentally friendly. The suggested RP-HPLC and TLC methods' green assessment profile, as well as the other technique utilizing the GAPI tool, are shown in Table 5.

#### Analytical GREEnness metric (AGREE)

Pena-Pereira has developed AGREE in June 2020, a downloadable greenness assessment software [40]. AGREE is based on the twelve fundamentals of GAC, SIGNIFICANCE. The final score in AGREE, is a fraction of a unit, going from zero to one. The generated pictogram is separated into twelve portions, with the ability to adjust the width of each component based on its significance. Each segment has a unique color scheme that ranges from dark green (= 1) to dark red (= 0). The circular pictogram's center contains the final score. The AGREE tool was made with basic principles in mind, such as inclusivity, input flexibility, simplicity, and yield clarity [55]. The tool was accessed through a link mentioned in AGREE publications [40, 56].

The proposed RP-HPLC and TLC approaches are greener than the previous reported method, according to GAPI, and AGREE assessment tools, the proposed methods produced greener GAPI quadrants. Additionally, the AGREE score (0.61) for RP-HPLC and (0.6) for TLC are greater than previously reported (0.46). While the proposed methods and the published one are equal in NEMI scoring, the methods have one non-shaded quadrant that related to hazardous quadrant and three quadrants were colored green, satisfying three NEMI criteria. The results indicated that the suggested techniques have a minimal impact on the environment (Table 5).

The suggested TLC approach was successfully applied in determining MIC and LDC in their synthetic mixtures and in their gel dosage form. While RP-HPLC method was successful to determine MIC, LDC and MTP in their gel dosage form beside DMA in presence of SAC without interfering with one another. The LOD of DMA by the proposed RP-HPLC method is 0.084 µg/mL and the MTP was revealed to be within acceptable limits (0.13%). The system suitability parameters were found to be satisfactory (Table 1). The developed methods were validated in accordance with ICH guidelines, to evaluate sufficient validation characteristics (Table 2). The specificity of the proposed chromatographic approaches was confirmed by laboratory mixtures analysis (Table 2). Furthermore, the methods' robustness was assessed by deliberate changes in some experimental circumstances and then calculating the %RSD (Table 2). The RP-HPLC method validity was confirmed using the standard addition technique (Table 3).

## Conclusion

Environmentally friendly, accurate, and sensitive TLC and RP-HPLC methods are presented. GAPI, NEMI and AGREE tools were used to evaluate the greenness of the methods. Additionally, we used the three tools to compare the suggested methods to the published one. The results demonstrate that the proposed methods have a decreased ecological impact. In general, evaluating the analytical methodologies' greenness should be included in method validation parameters. Additionally, prior to conducting practical trials in a lab, the sustainability of analytical techniques should be established to minimize the risk of chemicals being released into the environment. Furthermore, critical separation conditions in TLC, such as developing system and scanning wavelength all have been studied, in addition, flow rate, composition of mobile phase and the type of elution for RP-HPLC have also been examined.

Overall, our results indicate that the proposed TLC technique can be utilized to determine MIC and LDC. Consequently, it became necessary to shift to the RP-HPLC approach to analyze the toxic impurity; DMA, which was not separated by the TLC approach, along with the primary active ingredients MIC and LDC, and the excipient; MTP in bulk powder, mixtures, and a pharmaceutical formulation. The described methods could be employed for routine analysis in quality control labs.

## Data Availability

All data analyzed during this study are included in this published article and raw data are available from the corresponding author upon reasonable request.

## Abbreviations

GAC: Green analytical chemistry
LDC: Lidocaine hydrochloride
MIC: Miconazole nitrate
MTP: Methyl paraben
SAC: Saccharin sodium
DMA: Dimethyl aniline
TLC: Thin layer chromatography
HPLC: High performance liquid chromatography
ICH: International council for harmonisation
WHO: World health organization
BP: British pharmacopoeia
DAD: Diode array detector
NEMI: National environmental methods index
GAPI: Green analytical procedure index
AGREE: Analytical greenness metric
% RSD: Percentage relative standard deviation
EPA-TRI: Environmental protection agency's toxic release inventory
LOD: Limit of detection
LOQ: Limit of quantification
